# At the Intersection of Gut Microbiome and Stroke: A Systematic Review of the Literature

**DOI:** 10.3389/fneur.2021.729399

**Published:** 2021-09-24

**Authors:** Vishakha Sharma, Vaibhav Sharma, Shima Shahjouei, Jiang Li, Durgesh Chaudhary, Ayesha Khan, Donna M. Wolk, Ramin Zand, Vida Abedi

**Affiliations:** ^1^Kansas City University College of Osteopathic Medicine, Kansas City, MO, United States; ^2^Geisinger Commonwealth School of Medicine, Scranton, PA, United States; ^3^Geisinger Health System, Geisinger Neuroscience Institute, Danville, PA, United States; ^4^Department of Molecular and Functional Genomics, Geisinger Health System, Danville, PA, United States; ^5^Geisinger Health System, Geisinger Northeast Internal Medicine Residency, Wilkes Barre, PA, United States; ^6^Department of Laboratory Medicine, Geisinger Health System, Diagnostic Medicine Institute, Danville, PA, United States; ^7^Department of Public Health Sciences, College of Medicine, The Pennsylvania State University, Hershey, PA, United States

**Keywords:** stroke, TMAO, dysbiosis, nutrition, biomarker, microbiome

## Abstract

**Background:** Ischemic and hemorrhagic stroke are associated with a high rate of long-term disability and death. Recent investigations focus efforts to better understand how alterations in gut microbiota composition influence clinical outcomes. A key metabolite, trimethylamine N-oxide (TMAO), is linked to multiple inflammatory, vascular, and oxidative pathways. The current biochemical underpinnings of microbial effects on stroke remain largely understudied. The goal of our study is to explore the current literature to explain the interactions between the human gut microbiome and stroke progression, recovery, and outcome. We also provide a descriptive review of TMAO.

**Methods:** A systematic literature search of published articles between January 1, 1990, and March 22, 2020, was performed on the PubMed database to identify studies addressing the role of the microbiome and TMAO in the pathogenesis and recovery of acute stroke. Our initial investigation focused on human subject studies and was further expanded to include animal studies. Relevant articles were included, regardless of study design. The analysis included reviewers classifying and presenting selected articles by study design and sample size in a chart format.

**Results:** A total of 222 titles and abstracts were screened. A review of the 68 original human subject articles resulted in the inclusion of 24 studies in this review. To provide further insight into TMAO as a key player, an additional 40 articles were also reviewed and included. Our findings highlighted that alterations in richness and abundance of gut microbes and increased plasma TMAO play an important role in vascular events and outcomes. Our analysis revealed that restoration of a healthy gut, through targeted TMAO-reducing therapies, could provide alternative secondary prevention for at-risk patients.

**Discussion:** Biochemical interactions between the gut microbiome and inflammation, resulting in metabolic derangements, can affect stroke progression and outcomes. Clinical evidence supports the importance of TMAO in modulating underlying stroke risk factors. Lack of standardization and distinct differences in sample sizes among studies are major limitations.

## Introduction

The gut ecosystem encompasses various bacterial, viral, fungal, and protozoal species colonizing mucosal linings. The genetic density of the microbial genome outnumbers the human genome, with more than 3,000,000 microbial genes (1, 2). The integral role of the gut microbiome in metabolism, immune modulation, and vitamin production is governed by human cells' symbiotic relationship (3). Disturbances in balanced microbial structure, known as dysbiosis, result in increased pathogenic bacteria, and decreased commensal bacteria. For instance, in a comparison of oral and gut microbiomes, it has been shown that healthy aging individuals have an increased abundance of *Akkermansia* and *Erysipelotrichaceae* UCG-003 in their gut microbiome and, higher alpha diversity in their oral microbiomes; in that study, *Streptococcus spp*. was the only observed genus to have a significant reduction in its abundance in the oral and gut microbiomes of the healthy aging group (4).

Trimethylamine N-oxide (TMAO) is a key gut metabolite, derived from our diet, that has been shown to play a role in the development of various diseases. Mechanistic involvement of TMAO in the association between gut microbiome and stroke can provide potential therapeutic targets. The vast implications and effects of the gut microbiome in multi-disease processes make it difficult for research to be conducted to distinguish a single disease process. As the interdisciplinary topic of the gut microbiome and stroke is relatively novel, current investigations primarily focused on the microbial abundance in disease states are limited in providing functional or mechanistic information that may be vital in altering clinical outcomes.

Stroke, the fifth leading cause of death, affects more than 795,000 individuals in the United States every year and contributes to long-term disability, leading to both cognitive and ambulatory impairments (5). Stroke is broadly classified as ischemic and hemorrhagic. Risk factors for ishemic stroke include hypertension, diabetes, heart disease, hypercholesterolemia, smoking, and age. Ischemic stroke is classified into subtypes using the Trial of ORG 10172 in Acute Stroke Treatment (TOAST) classification, including large artery atherosclerosis, cardioembolic, small vessel occlusion, other determined cause, or undetermined cause (6). There is also evidence that polygenic risk scores can augment stroke subtyping (7).

An emerging role of the gut microbiome in stroke patients provides deeper insights regarding potential avenues to improve outcomes at a personalized level. Our systematic review discusses current clinical evidence on the human gut microbiome's involvement in ischemic stroke at different stages of disease progression, recovery, and management. We further examine the role of ischemic stroke's etiological classification as a potential modulator. Gaining a deeper understanding of stroke modulators can guide the development of targeted strategies based on an ischemic event's etiological features. Knowledge about stroke-associated gut pathogenic bacteria (8) along with the development of machine learning algorithms, capable of recognizing and analyzing microbial parameters, can enhance diagnostic procedures, allow early identification of stroke risks, and facilitate precise interventions for improved outcomes. In the following sections, we aimed to answer the following questions: (1) What are the key findings in current literature that suggests the influence of the human gut microbiome on at-risk and current stroke patients? (2) What characteristics of gut metabolite trimethylamine N-oxide make it a suitable target for stroke prevention and intervention? We conclude with a brief discussion and perspectives for future directions with a focus on actionable biomarkers for targeted therapeutic interventions.

## Methods

In phase I, we developed a comprehensive search strategy combining the three major themes of stroke, gut microbiome, and TMAO. The search string for stroke and gut microbiome were: (“Stroke”[Mesh] OR “Stroke, Lacunar”[Mesh] OR “Stroke Rehabilitation”[Mesh] OR “Infarction, Posterior Cerebral Artery”[Mesh] OR “Brain Stem Infarctions”[Mesh] OR “Infarction, Middle Cerebral Artery”[Mesh] OR “Infarction, Anterior Cerebral Artery”[Mesh] OR “Myocardial Infarction”[Mesh] OR “Anterior spinal artery stroke” [Supplementary Concept]) AND (“Microbiota”[Mesh] OR “Gastrointestinal Microbiome”[Mesh]) OR (stroke and microbiome). The search string for TMAO was: (“trimethyloxamine” [Supplementary Concept]) AND (“Diet”[Mesh] OR “Medication Therapy Management”[Mesh] OR Genetics). We included all published studies on these topics regardless of intervention target, approach, setting, and study design. We focused on human subject studies. PubMed database was searched and studies available between January 1, 1990, and March 22, 2020, were included. After performing the search, we manually screened the titles and abstracts for clinical applicability of gut microbiome in pre-stroke, stroke, and post-stroke stages.

In phase 2, we updated our search on May 1, 2020, and reviewed recent animal studies covering the underlying mechanisms. The following data were extracted from the included articles: study design, study setting, sample size, and limitations. Two review authors (ViS and VaS) extracted the data from eligible studies and the extracted data were reviewed for accuracy and relevance. The principal summary measure was the number of current article subtypes on the gut microbial abundance in stroke disease states. Studies with multiple study designs were counted as two separate studies for accurate synthesis. Data were imported into a table format for visualization. Missing data were not applicable as the study design data was apparent in the eligible studies. To better characterize the current literature on gut-microbiome, the range of sample sizes of the grouped study design was also calculated. Given the complexity and the non-comprehensive nature of the current literature, studies on gut microbiota and stroke were grouped based on clinical features such as stroke risk factors, stroke onset, and post-stroke recovery. Studies on TMAO were grouped based on the source, genetics, nutrition, comorbidities, medication, and mechanisms.

## Results

The search protocol regarding the gut microbiome and stroke resulted in 222 articles, from which 68 human studies were eligible for preliminary analysis, shown in Supplementary Tables 1, 2. Table 1 outlines the different study types, the number of articles in each study type, and the range of sample size. Five out of the 68 human subject articles had 2 study designs. Our preliminary analysis showed that the majority of articles in our present study were cohort studies (*n* = 27) and cross-sectional studies (*n* = 25); only one study was a clinical trial. The study design with the highest maximum sample size included cohort and metagenomic (human) studies.

**Table 1:** Summary of selected studies on gut microbiome and stroke.

**Study type**	**Number of articles**	**Sample size range**
Case control	8	[60–1,244]
Cohort	27	[50–3,359,653]
Clinical trial	1	[28]
Metagenomics (human)	11	[8–1,049,861]
Other (Cross sectional and Experimental)	26	[18–4,007]

*Five of the studies were classified into more than one study type due to their methodology*.

**Figure 1:**
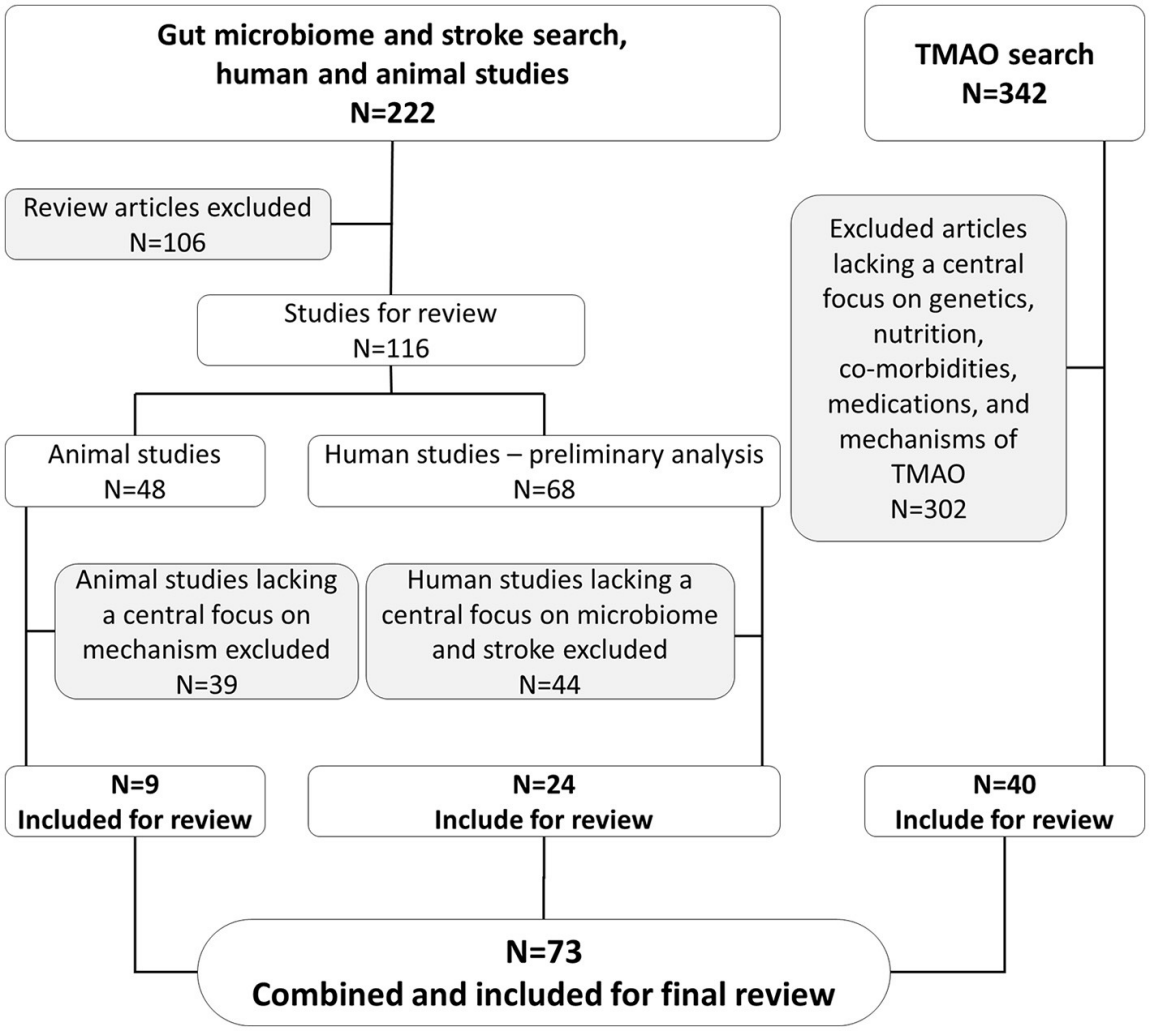
Schematic representation of inclusion and exclusion of articles in present study.

Among the preliminary 68 human subject studies, 24 articles were selected for further in-depth analysis and inclusion in our present study based on their pertinent findings for the gut microbiome and stroke. As human studies were limited in reporting underlying mechanisms, we included nine additional animal studies in our review. Our search was expanded to include 40 articles that focused on the effects of genetics, nutrition, comorbidities, medications, and mechanisms related to TMAO. In total, we included 73 articles in this review, as depicted in Figure 1.

### Gut Dysbiosis May Link Stroke Risk Factors to Stroke

#### Individuals With Stroke Risk Factors Exhibit Altered Gut Microbes

A review of the current literature at the intersection of the gut microbiome and stroke risk factors supports a better understanding of stroke management at a personalized level. Individuals with a high risk of stroke have increased opportunistic gut pathogens, increased lactate-producing bacteria, and decreased butyrate-producing bacteria (9).

Hypertension is a well-established risk factor for the development of the small-vessel occlusion (lacune) subtype of ischemic stroke, in which inflammatory cytokines are linked to recurrent vascular events (10). High systolic blood pressure is positively correlated with the presence of the genus *Prevotella* (11); findings were adjusted for age and gender. High dietary salt intake (≥2.3 g/d) was also associated with an increased relative abundance of genus *Prevotella*, family *Ruminococcaceae*, and genus *Bacteroides*, as shown in Table 2. The associated microbes statistically differed depending on short-term or long-term sodium consumption (11). Further, fecal microbiota transplantation studies performed on high-salt diet-fed mice to recipient germ-free mice resulted in higher blood pressure measurements following a low dose of angiotensin II (11). These findings suggest that the pathophysiology of microbiota-induced hypertension may be due to increased sensitivity of the angiotensin II receptor. Although the biochemical underpinnings remain poorly understood, interleukin-6 and interleukin-17 cytokines were found to be in greater systemic abundance in mice who received fecal microbiota transplantation from high-salt diet mice, compared to normal-salt diet mice (11).

**Table 2:** Gut microbial communities and their relative abundance change before stroke, during stroke, and after stroke event.

**Stages of stroke**	**Changes in the abundance**	**Gut microbial communities**
PRE-STROKE	Increase	Gut opportunistic pathogens, lactate producing bacteria, *Ruminococcaceae*, and *Peptococcaceae* as well as *Bacteroides, Prevotella, Clostridium, Escherichia, Enterobacter, Acinetobacter*, and *Proteus* species
	Decrease	Butyrate-producing bacteria, *Faecalibacterium prausnitzii*
STROKE ONSET	Increase	*Helicobacteraceae, Neisseriaceae, Ruminococcaceae_UCG_005, norank_p_Flavobacteriaceae, norank_p_Parcubacteria, Gammaproteobacteria, Proteobacteria*, and short chain fatty acid producing bacteria,as well as *Odoribacter, Akkermansia, Victivallis, Enterobacter, Megasphaera, Desulfovibrio, Acinetobacter*, and *Acidovorax* species, and *N. polysaccharea*
	Decrease	*Genus Bacteroidia* and *Faecalibacterium, Bacteroides spp.*, and *Prevotella spp*.
POST-STROKE	Increase	*Carnobacteriaceae, Streptococcaceae, Granulicatella, Streptococcus*, and *Fusobacterium spp.*, as well as *G. adiacens*
	Decrease	Short chain fatty acids

Atrial Fibrillation (AF) serves as a significant risk factor for cardioembolic ischemic strokes. Individuals with AF exhibit gut microbial imbalance and have similar taxonomic profiles (*Bacteroides, Prevotella*, and *Faecalibacterium* genera) but distinct profiles from non-AF individuals (12, 13). AF individuals had similar microbiome diversity and microbial structures that differed from controls (13). Limitations in these findings include a lack of consideration for dietary and physical activity effects on atrial fibrillation.

Metabolic syndrome pathology consists of vascular risk factors and metabolic derangements such as central obesity, dyslipidemia, hyperglycemia, and hypertension. Metabolic syndrome promotes the development of atherosclerosis and increases the risk of stroke. Patients with metabolic syndrome show a significant positive correlation between plasma TMAO concentrations and unclassified members of the order *Clostridales*, and a negative correlation with *Fusobacterium prausnitzii* (14). The link between metabolic abnormalities and gut microbial structure showed that higher glutamine levels were associated with increased unclassified *Clostridales*, while higher acetate levels were associated with phylum *Tenericutes* and family *Christensenellaceae*. Co-occurrence network analysis revealed that *Clostridales, Tenericutes, Methanobrevibacter*, and *Christensenellaceae* were positively correlated with metabolites acetate, glutamine, and polyunsaturated fatty acids, while *Blautia* were negatively correlated with acetate (14). Metabolic syndrome has been found to correlate with specific microbial communities and metabolites, which may indirectly impact stroke events.

Isolated bacterium transplantation of *Enterobacter cloacae B29* from a morbidly obese patient into germ-free mice, followed by a high-fat diet, induced obesity, and insulin resistance in mice models (15). In addition, bacterium-derived endotoxin load showed a causative relationship with inflammatory conditions (15). Anti-diabetic and anti-inflammatory medications affect the gut microbiota. For instance, polyphenol resveratrol given to obese Caucasians improved glucose homeostasis and insulin resistance along with a reduction in microbial diversity and an increase in *Akkersmansia muciniphila* (16). Similar results were not observed in obese African Americans, Afro-Latinos, and African American/Asian; indicating that polyphenol resveratrol intervention may exhibit ethnicity-dependent effects on glucose metabolism.

Diabetes mellitus is recognized as a major risk factor for both large artery atherosclerosis and small vessel disease etiology of stroke. Patients with type 2 diabetes and chronic kidney disease, prevalent risk factors for stroke, have an increased abundance of trimethylamine producing bacteria, including *Clostridium spp., Escherichia spp., Enterobacter spp., Acinetobacter spp.*, and *Proteus spp*. that belong to phylum Firmicutes and Proteobacteria. In comparison with healthy individuals, diabetic patients have higher serum levels of TMAO (17). Higher serum TMAO levels in diabetic patients contributed to widespread endothelial dysfunction, inflammation, and increased gut permeability compared to healthy counterparts (17). In addition, increased plasma TMAO in these patients correlates with plasma TNF-α, interleukin-6, zonulin gut permeability marker, endothelin-1, and lipopolysaccharide (17).

A cerebral autosomal dominant arteriopathy with subcortical infarcts and leukoencephalopathy (CADASIL), a rare genetic disease that can lead to recurrent lacunar and small vessel stroke, was shown to have significant taxonomic differences in gut microbiota compared to controls, suggesting the role of intestinal microbiota on the pathophysiology of CADASIL. In CADASIL patients with a history of stroke, there was a decrease in *Phascolarctobacterium* and *Paraprevotella* compared to patients without a history of stroke (18, 19).

Hemorrhagic stroke is a brain bleed that occurs due to ruptured blood vessels and can result from trauma, tumors, genetic malformations, hypertension, diabetes, and smoking. There are fewer investigations on the pathophysiological interaction between intestinal microbiota and hemorrhagic stroke compared to ischemic stroke. Nonetheless, intestinal microbiota has been implicated in the developmental pathogenesis of neurovascular abnormalities that result in hemorrhagic strokes, such as cerebral cavernous malformation (20). The lipopolysaccharide component of gram-negative bacteria from the gut localizes to the systemic circulation, interacts with Toll-like 4 endothelial receptor, and induces activation of MEKK3-KLF2/4 signaling (20). Further, genetic mutations in *PDCD10*, which arise from excess MEKK3 signaling via lipopolysaccharide from the gut microbiome, contribute to cerebral cavernous malformation in a mouse model (21).

#### Gut Metabolite TMAO Exacerbates Stroke Risk Factors

TMAO is a key gut metabolite that increases after the consumption of animal products. Higher levels of TMAO are associated with increased risk of major cardiovascular events which they defined as death, myocardial infarction, or stroke (22). Plasma TMAO is measured using stable isotope dilution liquid chromatography tandem mass spectrometry and normal plasma TMAO levels are 0.5–5 μM. Further studies trying to understand the prognostic value of TMAO showed that in patients who underwent cardiovascular surgery, high serum TMAO was associated with an increased number of infarcted coronary arteries, after adjusting for age, sex, body mass index, chronic kidney disease, hypertension, dyslipidemia, and cerebrovascular disease (23). The authors concluded that TMAO may be a useful biomarker in providing clinical utility in risk stratification among subjects suspected of an acute coronary syndrome. To better understand the mechanistic link between TMAO and thrombotic risk, the research group also showed in animal studies that TMAO enhances platelet hyperactivity and responsiveness. Additionally, TMAO precursor trimethyllysine, has been shown to be associated with both short-term and long-term cardiovascular events. Trimethyllysine levels in patients with suspected acute coronary syndrome were associated with major cardiac events, including stroke at 1 and 6 months, independent of cardiovascular risk factors or renal function (24).

Nie et al. showed, using a nested case-control study, that higher levels of TMAO were associated with increased risk of stroke in a hypertensive population when adjusted for choline, L-carnitine, and baseline systolic blood pressure (25). Another study focusing on type 1 diabetics showed that higher plasma concentrations of TMAO were predictive of mortality and cardiovascular disease. Stroke was not significantly associated with higher TMAO concentrations in type 1 diabetics when adjusted for cardiovascular risk factors (26). Limitation of this study included the one-time TMAO level measurement and the high intraindividual variability in TMAO levels.

Importantly, inconsistencies still exist in the association between TMAO precursors and cardiovascular disease risk. For instance, Jia et al. (27) show that genetically predicted higher TMAO is not associated with higher odds of cardiometabolic disease traits (atrial fibrillation, coronary artery disease, myocardial infarction, stroke) after Bonferroni correction. They showed that type 2 diabetes and chronic kidney disease result in higher TMAO levels and that cardiovascular disease may be a result of confounding or reverse causality. An important study limitation was the exclusion of additional variants of selected genome-wide SNPs that were used for Mendelian randomization (27). Taken together, TMAO may link stroke risk factors with various health conditions, including stroke, vascular inflammation, and other vascular events.

### Microbial Communities During Ischemic Stroke Event

The gut microbiota in ischemic stroke and intracerebral hemorrhage stroke patients differ in α-diversity (within-individual diversity), β-diversity (between-individual diversity), and taxonomic summary compared to healthy controls (28). A small cohort of 30 ischemic stroke patients had enriched *Odoribacter, Akkermansia, Ruminococcaceae_UCG_005, norank_p_Flavobacteriaceae, norank_p_Parcubacteria*, and *Victivallis* as well as increased short-chain fatty acid-producing bacteria, compared to 30 healthy controls who had enriched *Anaerostipes* and *Ruminiclostridium_5* (29). Similarly, a small cohort of 10 patients with cerebral infarction had an increased *Gammaproteobacteria* and decreased *Bacteroidia*. The presence of *Gammaproteobacteria* was positively correlated with apolipoprotein E. In contrast, *Bacteroidia* was negatively correlated with apolipoprotein E levels (30). Patients with large-artery atherosclerotic ischemic strokes and transient ischemic attacks had depleted *Bacteroides, Prevotella*, and *Faecalibacterium* but had an increased density of *Enterobacter, Megasphaera*, and *Desulfovibrio*.

**Figure 2:**
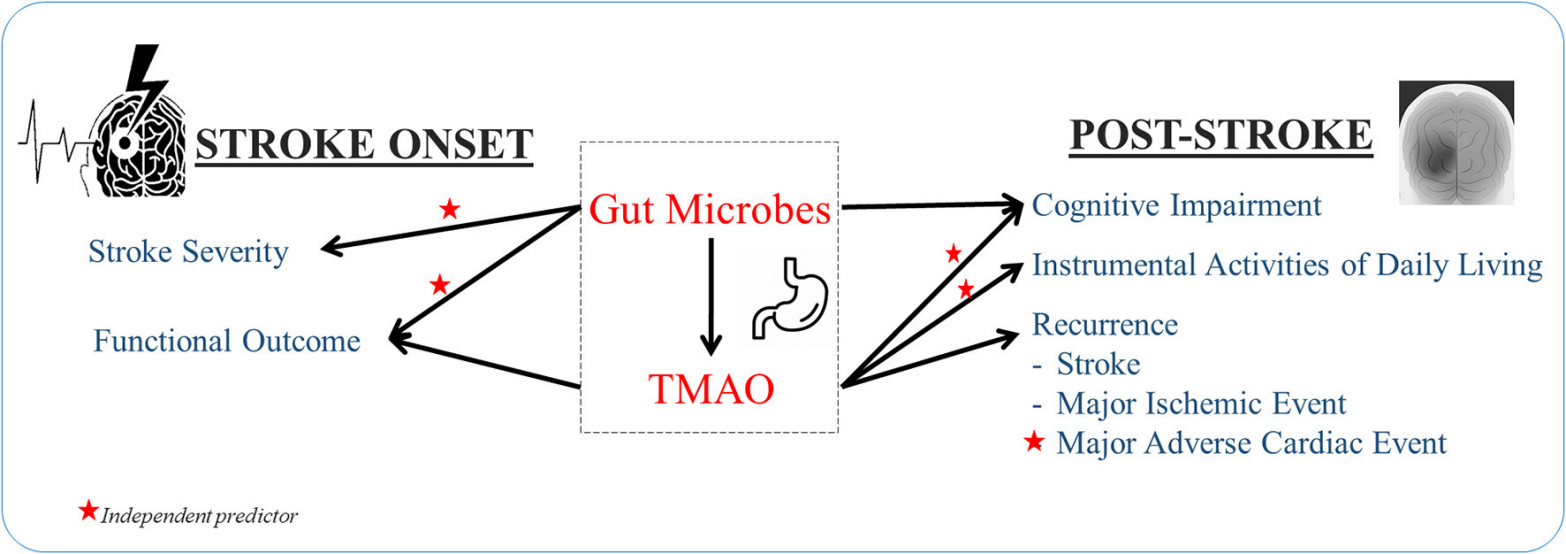
Gut dysbiosis and plasma TMAO independently predict stroke features.

*Proteobacteria* was abundant in severe stroke patients compared to patients with mild stroke, as shown in Figure 2 (31). Stroke patients exhibited low TMAO levels compared to controls; however, the article lacked age- and sex- matched controls and measured levels of TMAO 24 h after hospital admission, in which the stroke physiology or treatment may have impacted TMAO levels (31). A more recent study by the same group showed that TMAO levels in AIS patients decrease 24 h after treatment. However, baseline TMAO levels were higher in AIS patients compared to controls. After 7 days post-treatment TMAO levels were lower in AIS patients compared to controls (32). Based on differential genera in large-artery atherosclerotic ischemic stroke, a stroke dysbiosis index was developed and independently correlated with stroke severity and stroke outcome at discharge (33). Further, patients who underwent carotid endarterectomy due to previous strokes or transient ischemic attack had an abundance of *Helicobacteraceae, Neisseriaceae, Acinetobacter, Acidovorax*, and *N. polysaccharea* compared to asymptomatic atherosclerotic patients who had an abundance of *Porphyromonadaceae, Bacteroidaceae, Micrococcaceae*, and *Streptococcaceae* (34). However, another study found similar bacterial profiles, dominated by *Proteobacteria* and *Actinobacteria*, between asymptomatic patients and symptomatic patients with cerebral ischemia (including stroke, transient ischemic attack, and amaurosis fugax) (35). Both of these studies defined symptomatic plaques obtained from patients with symptomatic atherosclerosis. However, the first study defined asymptomatic plaques from patients who died due to non-atherosclerosis causes, while the second study defined asymptomatic plaques from living asymptomatic patients.

In addition to alterations in gut microbes during a stroke, plasma TMAO levels are also affected. High levels of TMAO in plasma are associated with early neurological deterioration, defined as a two or more point increase in the National Institutes of Health Stroke Scale (NIHSS) in 3 days, in patients with thrombotic, lacunar, embolic, or other ischemic stroke types (36). Despite having higher initial baseline TMAO levels, ischemic stroke patients had decreased plasma TMAO levels 24 h and 7 days after treatment was administered compared to controls (32). However, there is not sufficient evidence to corroborate causality. As ischemic stroke occurs concomitantly with gut dysbiosis and altered plasma TMAO levels, the microbiome is likely involved in stroke occurrence.

### Gut Metabolite in Post-ischemic Stroke Events

#### The Contribution of the Microbiome in Stroke Recovery Remains Largely Understudied

The alterations of microbiome diversity during stroke recovery and how various therapies alter the structure remain largely understudied. Further advancements in this topic may lead to the improvement of personalized treatment modalities. A study performed on patients recovering from an acute stroke episode showed differences in both oral and gut microbiomes structure depending on whether patients received oral or tube feedings. Opportunistic pathogens such as *Corynebacterium striatum* and *Streptococcus agalactiae* were found in higher proportion, and dominant genera such as *Streptococcus spp*. and *Veillonella spp*. were found in lower densities during enteral feedings when compared to oral feedings (37). These results support the notion that oral indigenous microbiota is altered to allow for opportunistic pathogens to thrive in enteral feedings compared to oral feeding during stroke recovery. The pathophysiology behind these findings can be attributed to various factors such as decreased mechanical clearing with mastication and reduction in salivary flow. More long-term studies are needed to corroborate the overall biological effect of these microbiome alterations as well as more in-depth studies considering the various subtypes of ischemic stroke.

In microbiome-related studies, another relevant factor for consideration is the pattern of co-occurrence. The genetic profiles of microbiomes can be utilized to construct networks to quantify and visualize varying degrees of co-occurrences, which could also indicate their potential interactions. Simpler co-occurrence networks were observed more frequently in oral and gut microbiomes in oral feeding compared to enteral feedings. The clustering coefficients' values, which quantify the abundance of connections in an ecological network for microbiome interaction analysis, showed a declining trend—from 0.44 (oral feeding) to 0.375 (tube feeding)—within all networks in the oral microbiome. In the gut microbiome, the trend was also decreased—from 0.542 (oral feeding) to 0.467 (tube feeding) (37).

These observations highlight that patients' dysbiosis in the post-stroke period is potentially decreased with oral feedings compared to enteral feedings. Although evidence is still limited, current literature points to the importance of food intake by the oral route during stroke recovery regardless of stroke etiology.

There is a lack of understanding about the human gut microbiome in stroke recovery. However, a study in animals identified the pro-regenerative effects of short-chain fatty acids (SCFAs) in modulating stroke recovery. SCFAs promote microbiota activation and behavioral recovery, measured via motor deficits of the affected forelimb, and lead to post-stroke neuronal plasticity, defined as dendritic spine density of pyramidal cells (38). These findings suggest that SCFA can play a vital role in stroke recovery by reducing the invasion of immune cells or cytokine secretion in the brain. Further, ischemic stroke is associated with decreased SCFA levels and fecal microbiota transplants rich in SCFA serve as effective treatments (39). Future studies are needed to explore the human gut microbiome and SCFAs in the stroke recovery process.

#### Gut Metabolite TMAO Is Associated With Post-stroke Complications

A study performed in patients with acute ischemic stroke showed that elevated baseline TMAO levels measured at day 2 and day 7 post-stroke were associated with increased 90 day and 12-month major ischemic complications such as myocardial infarction, death, or recurrent ischemic stroke as well as unfavorable functional outcomes as indicated by modified Rankin scale (mRS) ≥3 (32). No significant differences were observed among patients with different TOAST criteria. Studies performed on high-choline-fed mice have shown to increase macrophage and scavenger CD36 receptors to promote the development of atherosclerosis (40). These findings were reversed with broad-spectrum antibiotic suppression of intestinal microbiota (40). The underlying mechanism provides an understanding of how high TMAO levels may result in worsening atherosclerosis in patients recovering from stroke with large-artery atherosclerotic subtype. The specific mechanism of major ischemic complications in other subtypes of ischemic stroke is unclear. TMAO's effect on vascular inflammation, endothelial dysfunction, oxidative stress, and signal inhibition may also play a role. Other studies examining long-term data (up to 3 years follow-up) support the association between TMAO and stroke outcomes. A study examining thrombotic, lacunar, and embolic post-stroke patients showed that high TMAO levels had higher major adverse cardiovascular events 3 years later than counterparts with low TMAO levels (41). TMAO may have consequential long-term effects, thus potentially providing clinicians a window of opportunity for intervention. Further research will be needed to evaluate whether decreased TMAO levels or decreased microbiome-associated choline metabolism has clinical value. TMAO could provide prognostic value that can aid care providers in identifying the risk of recurrence and providing timely interventions for secondary prevention. Furthermore, TMAO may also be integrated, along with traditional risk factors, into predictive models to identify patients at higher risk for recurrence and facilitate the implementation of more targeted preventive measures.

**Figure 3:**
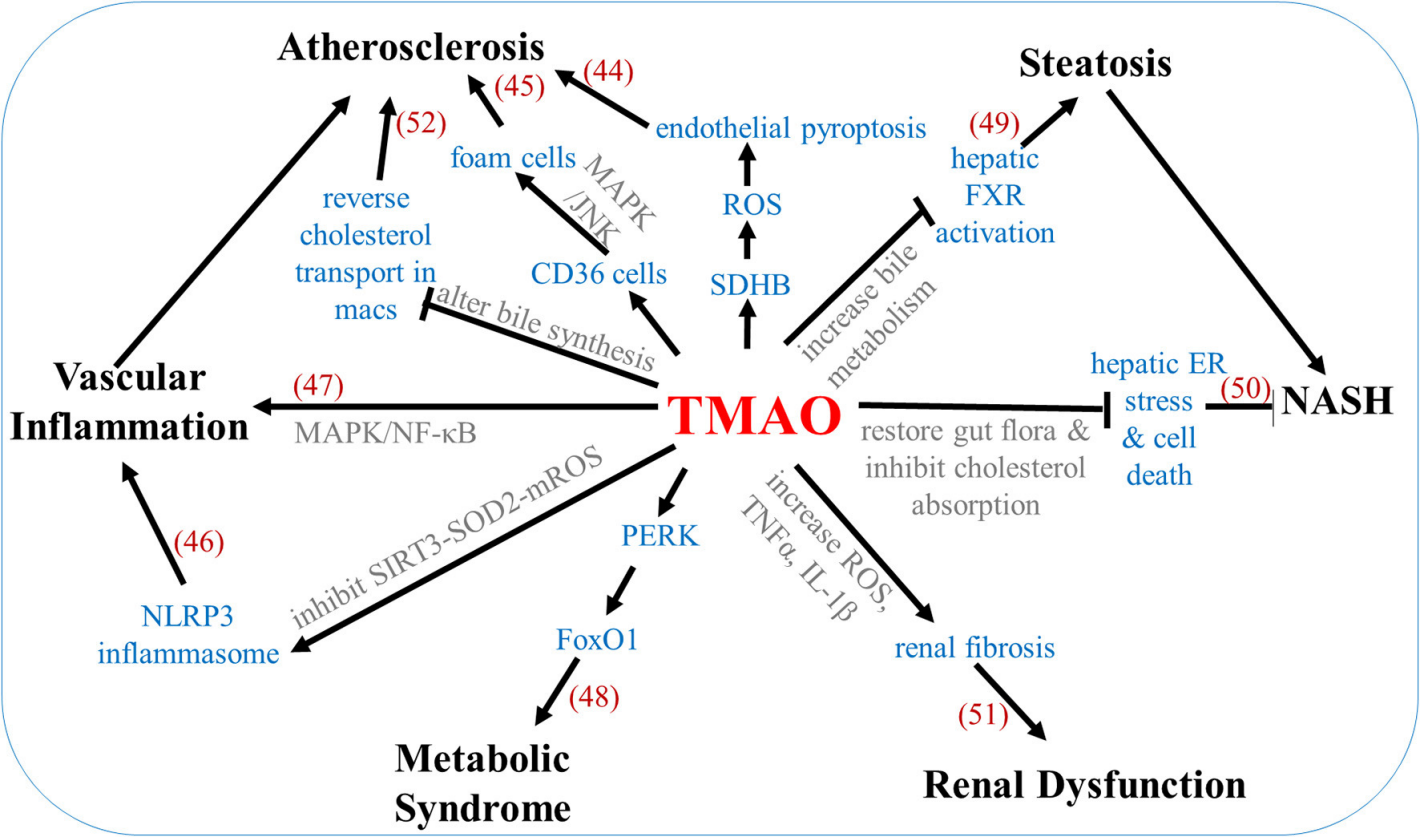
Mechanisms underlying TMAO-associated complications. Numbers in parentheses indicate supporting references.

TMAO can also have an indirect effect on recurrent stroke through alterations in the risk factors. TMAO increases osmotic pressure by influencing the body's counter-regulatory mechanism to promote water resorption via the TMAO-AVP-AQP-2 axis, effectively increasing the mean arterial blood pressure, and leading to hypertension (42). TMAO is also seen to impair glucose tolerance and insulin signal pathways. High-fat diet mice which also received 0.2% TMAO for 4 weeks were seen to have increased insulin levels, increased inflammatory cytokine MCP-1, and decreased anti-inflammatory cytokine IL-10 in the adipose tissue when compared to high-fat diet mice without 0.2% TMAO (43). Figure 3 shows the underlying mechanisms involved in TMAO-linked complications (44–52).

### TMAO as an Actionable Metabolite

TMAO is derived from our diet through phosphatidylcholine, L-carnitine, and betaine, shown in Table 3. Gut microbes convert dietary nutrients into intermediate trimethylamine, which undergoes oxidation by hepatic enzyme flavin-containing monooxygenases to produce TMAO. The impact of genetics on TMAO is largely determined by enzyme flavin-containing monooxygenases 3, which is responsible for 90% of trimethylamine oxidation (53). *FMO3* overexpression in mice increases plasma TMAO levels while silencing *FMO3* decreases TMAO levels. In humans, males express hepatic *FMO3* in lower quantities than females, possibly due to down-regulation via androgen (53). Enzyme variants are associated with TMAO and kidney function decline in individuals with chronic kidney disease (54). Flavin-containing monooxygenases 3 impacts TMAO levels, platelet responsiveness, and thrombosis potential (55). Knockout studies have shown TMAO and thrombosis reduction along with effects on lipid and glucose metabolism (43, 44). Manipulation of phosphatidylethanolamine N-methyltransferase, an enzyme involved in choline metabolism, has also been shown to impact TMAO levels (56).

**Table 3:** Impact of genetics, nutrition, medications on TMAO, and associated comorbidities.

**Source**	**Genetics/Epigenetics**	**Nutrition**	**Comorbidities**	**Medications**
Phosphatidylcholine	Flavin-containing monooxygenases 3	**Increases TMAO** Omnivore, fat-based diet rich in saturated fat, red meat, lean seafood, salmon, eggs, non-fermented dairy products, salt, double the daily amount of protein, Paleolithic diet	HIV Diabetes Preeclampsia Gestational diabetes Polycystic ovary syndrome Coronary artery disease Coronary heart disease Plaque rupture in STEMI Thrombus in atrial fibrillation Chronic kidney disease	**Increases TMAO** Antiretroviral therapy β glucuronidase inhibitor
L-carnitine	Phosphatidylethanolamine N-methyltransferase	**Decreases TMAO** Hypocaloric diet & exercise, pistachio, resveratrol, pterostilbene, oolong tea extract, citrus peel polymethoxyflavones, fish oil		**Decreases TMAO** Aspirin Antibiotics Meldonium Clopidogrel Choline analog Guggulsterone
Betaine	MicroRNA 146a-5p	**No effect** Probiotics, inulin fiber, tea, or cocoa flavanol		

While genetics play a role in determining TMAO levels, dietary nutrients intake is also an important contributor. Studies suggested that animal-based diets rich in saturated fat lead to increased plasma levels of TMAO (57–59). Consumption of lean seafood (60), salmon (61), eggs (62), non-fermented dairy products (63), increased intake of salt, increased intake of protein (64), and adherence to a Paleolithic diet (65) result in higher TMAO. On the other hand, pistachio intake (66), hypocaloric diet, and exercise (67) have decreased TMAO in humans. Consumption of resveratrol (68), pterostilbene (69), oolong tea extract and citrus peel polymethoxyflavones (70), and fish oil (71) have reduced TMAO in mice. In contrast, intake of probiotics, inulin fiber (72), and tea or cocoa flavanol (73) did not alter TMAO levels. These findings suggest that nutrition can play an important role in determining concentrations of gut metabolite TMAO. Nutrition therapy and personalized diets can serve as a potential intervention for patients exhibiting gut dysbiosis or TMAO-linked conditions. In addition to diet, medications may also have an impact on TMAO levels. In humans, TMAO is suppressed by broad-spectrum antibiotics (74), aspirin and clopidogrel (32), and meldonium (75). In mice, TMAO is suppressed by a choline analog (3,3-dimethyl-1-butanol) (76) and guggulsterone (farnesoid X receptor antagonist) (77). TMAO enhancing medications include antiretroviral therapy in HIV patients (78) and β glucuronidase inhibitor (glucaro 1, 4 lactone) in rats (79). Additional investigations are needed to examine how common medications given to patients with stroke or its risk factors may impact the TMAO level and potentially lead to a poor outcome in these patients.

## Discussion and Future Prospectives

Our results showed that human gut microbial communities can be involved in different stroke stages, from facilitating the progression of stroke in the presence of its risk factors, to the recurrence of stroke. We identified altered gut microbes in pre-stroke, stroke onset, and post-stroke phases, which may serve as potential clinical biomarkers. The current understanding of the gut microbiome revolves around the large artery atherosclerosis subtype, probably due to its high prevalence compared to other etiologies. However, the microbiome's vast effects can play a role in the pathogenesis of all stroke etiologies.

Our findings also demonstrate that TMAO is a powerful metabolite that plays an essential role in the association between microbiome and stroke and may serve as an actionable target. As there is new evidence that polygenic risk scores can be used to augment stroke subtypes (7), microbiome composition can also be an additional layer of information that can be used to better inform care providers when planning individualized care paths for their patients to improve outcome. Taken together, the gut microbiome plays an important role in stroke and can transform how stroke and its subtypes are diagnosed and treated.

Gut dysbiosis and gut metabolite TMAO, involved in different stages of stroke, may contribute to stroke development and associated outcomes. These findings demonstrate the importance of maintaining a healthy gut by preserving the normal balance of commensal and pathogenic bacteria and optimal TMAO levels to reduce the risk of stroke incidence. Implementation of personalized solutions focused on restoring microbial communities and lowering TMAO through the diet may improve the quality of life for individuals living with morbid conditions. Dietary interventions can change gut microbial composition from a disease state to a homeostatic state.

Prebiotics are dietary fiber-derived, non-digestible carbohydrates that selectively stimulate the growth or activity of certain colon-resident bacteria (80). For instance, prebiotics can enhance the growth of anti-inflammatory bacteria *Lactobacillus* and *Bifidobacterium* (81). Examples of prebiotics include inulin, human milk oligosaccharides, fructo-oligosaccharides, galacto-oligosaccharides (82). Sources of prebiotics include soybeans, raw oats, unrefined wheat, and unrefined barley (83). Diets rich in prebiotics can shift the microbiome composition toward increased commensal bacteria, thereby promoting host health.

While prebiotics enhances intrinsic microbial communities, probiotics are exogenous live microorganisms, taken orally, that confer health and benefit to the host via modulation of intestinal microflora and inhibition of pathogen colonization (84). Probiotics can restore the proper balance of gut microbiota, exert metabolic effects, and enhance immunomodulatory pathways (85). Probiotics belonging to the genera *Lactobacillus, Bifidobacterium, Lactococcus, Streptococcus, and Enterococcus* (85), are found in fermented foods such as cheese, kefir, bushera, salami, kimchi, sauerkraut, olives (86). Incorporating prebiotics, probiotics, or both (known as synbiotic) into a personalized diet may attenuate the development of pathogen-dominant microbiome-associated diseases, especially in patients with bacterial translocation or inflammation. For instance, the probiotic species *Bifidobacterium animalis* can potentially aid in the reversal of bacteremia and lower inflammation in type 2 diabetes, a common risk factor for stroke (87).

The gut microbiome exhibits inter-individual variability in age, sex, ethnicity, and disease states (88); therefore, similar dietary recommendations given to different individuals may not effectively improve their dysbiosis. Care pathways could be designed for individual variations when incorporating personalized nutrition therapies with specific probiotic strains or prebiotic consumption.

In addition to diets focused on restoring gut microbiota as a whole system, personalized nutrition therapies could specifically target and lower TMAO levels, which are often linked to numerous disease states. Studies have reported that dietary precursors can predict TMAO levels (89) and precursor phosphatidylcholine can heighten TMAO levels (74). Increased precursor choline or betaine are further associated with increased risk for major adverse cardiac events (90). These findings suggest that decreased consumption of TMAO precursors may lower TMAO levels and may improve its associated conditions. TMAO precursors are found in red meat and egg yolk. Starting a dietary approach to stop hypertension (DASH) (91), vegetarian diet, or Mediterranean diet may reduce TMAO, especially for patients with high plasma TMAO levels (92). As a long-term diet is associated with both the metabolome and microbiome (93), persistent dietary changes, focused on improving gut health, may reduce stroke risk and promote secondary prevention. Personalized nutrition strategies account for the personalized microbiota response and personalized host response to a specific diet or nutrients.

Machine learning-based personalized dietary interventions, developed based on the patient's microbiome composition, clinical parameters, genetics, lifestyle patterns, and personal goals, can be designed to impact the microbiome, metabolome, and gut physiology (94–97). Patient TMAO levels can be integrated into the model to tailor a diet focused on lowering TMAO and mitigating the risk of developing related conditions. Quantification of various biomarkers such as choline, carnitine, betaine, trimethylamine, and TMAO in the clinical setting can be a convenient and effective way to identify actionable risk factors for stroke recurrence or poor outcome and facilitate the development of technologies with quantitative features for targeted interventions. A well-designed and validated model can analyze patient profile, including patient's altered gut microbes, increased TMAO levels, systemic inflammatory markers, medication history, and traditional stroke risk factors obtained from the electronic health record, and estimate the risk of recurrence or poor outcome. Such models can also integrate other non-clinical variables such as nutrition patterns for a more precise and personalized assessment and recommendations (95, 98).

The gut microbiome contributes to ischemic stroke progression and includes large vessel subtypes and small vessel pathophysiology. Our findings in the present study are considered preliminary. Lack of standardization between articles in an interdisciplinary field limits applicability to the clinical setting. Factors such as lifestyle, physiologic traits, alcohol consumption, and bowel movements are confounding variables that should be considered in future studies. Vujkovic-Cvijin and colleagues provide a list of important confounding factors to consider when designing such studies on this topic (99). One of the limitations in our present study is the inclusion of articles with small sample sizes, which warrant additional large-scale investigations. Reports with large sample sizes included genomic databases while articles with small sample sizes included investigations performed in hospital or university settings. Further, the diversity and composition of gut microbiota lead to complex network analysis, and articles in our present study primarily examined increases or decreases in microbiota composition. Studies included here did not incorporate the concept of functional redundancy-induced stability of gut microbiota due to the multiple functioning capacity of different microbes. Future studies need to examine functional changes rather than compositional changes of the gut microbes to demonstrate how the microbes induce changes within the human body. Current biochemical knowledge on microbiome's effects on stroke was extrapolated from animal studies that were similar but not specific to gut influences on stroke. Further, our review yields a low number of articles on hemorrhagic stroke compared to ischemic stroke; the latter could be in part since hemorrhagic stroke is associated with higher morbidity, mortality, and a lower prevalence than ischemic stroke. Additional investigations are needed to explore the gut microbiome alterations that occur during a hemorrhagic stroke. Some of the articles included in our present study were specific to particular ethnic populations, such as Caucasians and Asians. The latter may provide insight into how genetics or environment (nutrition and physical activity) may differentially impact gut microbiomes across ethnicities, which is important for better patient representation and addressing health disparity, and the development of personalized therapies. Limitations in our review process include selecting published articles in English and using only one database (Pubmed).

In conclusion, our findings support the involvement of the gut microbial landscape and key metabolite TMAO in stroke risk factors, occurrence and recurrence of ischemic stroke, and functional outcomes after stroke. Future human subject studies are needed to elucidate specific functions and underlying mechanisms of gut microbes that play a role in the pathophysiology of stroke. Translating this knowledge into clinical practice can aid care providers in delivering personalized and preventative care to stroke patients efficiently.

## Data Availability Statement

The original contributions presented in the study are included in the article/Supplementary Material, further inquiries can be directed to the corresponding author/s.

## Author Contributions

VA and RZ: conception of the work. ViS, VaS, VA, and AK: data collection. VA, RZ, ViS, VaS, AK, DC, SS, JL, and DW: data analysis and interpretation. ViS, VaS, and VA: drafting the article. ViS, VaS, DW, JL, SS, DC, RZ, and VA: critical revision of the article. All authors approved the final version of the article.

## Conflict of Interest

Unrelated to this study, VA had financial research support from the National Institute of Health (NIH) grant No. R56HL116832 sub-awarded to Geisinger during the study period. RZ had financial research support from Bucknell University Initiative Program, Roche—Genentech Biotechnology Company, the Geisinger Health Plan Quality fund, and receives institutional support from Geisinger Health System during the study period. The remaining authors declare that the research was conducted in the absence of any commercial or financial relationships that could be construed as a potential conflict of interest.

## Publisher's Note

All claims expressed in this article are solely those of the authors and do not necessarily represent those of their affiliated organizations, or those of the publisher, the editors and the reviewers. Any product that may be evaluated in this article, or claim that may be made by its manufacturer, is not guaranteed or endorsed by the publisher.
