# Supplementary material for: At the Intersection of Gut Microbiome and Stroke: A Systematic Review of the Literature
Source: Front Neurol. 2021 Sep 24;12:729399. doi: 10.3389/fneur.2021.729399 (PMC8498333; doi:10.3389/fneur.2021.729399)
Supplement: Supplementary file 1 [file Table_1.docx]

**Supplemental Table S1.** Preliminary analysis of study design and sample size on human subject studies.

| PMID | Study Design | Sample Size | Included Studies (Y/N) | Reference |
| --- | --- | --- | --- | --- |
| 32189845 | Case Control | 96 | N |  |
| 31620923 | Case Control | 65 | Y | 4 |
| 31426765 | Case Control | 60 | Y | 29 |
| 30354996 | Case Control | 1244 | Y | 25 |
| 29080862 | Case Control | 980 | N |  |
| 26597155 | Case Control | 553 | Y | 31 |
| 26536303 | Case Control | 92 | N |  |
| 26413854 | Nested Case Control, Cross Sectional | 215, 210 | N |  |
| 31420758 | Cohort | 256 | N |  |
| 31382927 | Cohort | 26117 | N |  |
| 31332666 | Cohort | 225 | N |  |
| 31216010 | Cohort | 36429 | N |  |
| 31151471 | Cohort | 98 | Y | 28 |
| 31123156 | Cohort | 1159 | Y | 26 |
| 30658186 | Cohort | 3359653 | N |  |
| 30553164 | Cohort | 859 | N |  |
| 30100156 | Cohort | 316 | N |  |
| 29976769 | Cohort | 671 | N |  |
| 29678946 | Cohort | 213661 | N |  |
| 28543778 | Cohort | 50 | N |  |
| 28407784 | Cohort | 531 | Y | 14 |
| 31049589 | Cohort | 530, 1683 | Y | 24 |
| 28077467 | Cohort | 530, 1683 | N |  |
| 27515213 | Cohort | 1000 | N |  |
| 26868510 | Cohort | 326 | N |  |
| 26731277 | Cohort | 161,490 | N |  |
| 25522313 | Cohort | 219 | N |  |
| 24497336 | Cohort | 3903 | N |  |
| 20453665 | Cohort | 653 | N |  |
| 19053919 | Cohort | 161 | N |  |
| 15699278 | Cohort | 657 | N |  |
| 29702430 | Cohort, Cross Sectional | 316 | N |  |
| 31590480 | Cohort, Cross Sectional | 313 | Y | 41 |
| 23614584 | Cohort, Experimental | 4007, 40 | N |  |
| 31929168 | Cohort, Experimental | 65, 18 | N |  |
| 30873501 | Clinical Trial | 28 | Y | 16 |
| 32188747 | Metagenomics (human) | 100 | Y | 13 |
| 31996208 | Metagenomics (human) | 1033 | N |  |
| 31956606 | Metagenomics (human) | 8 | Y | 37 |
| 31777426 | Metagenomics (human) | 31 | Y | 18 |
| 31308384 | Metagenomics (human) | 1079 | N |  |
| 31164901 | Metagenomics (human) | 150 | N |  |
| 29930110 | Metagenomics (human) | 1049861 | N |  |
| 29900345 | Metagenomics (human) | 20 | N |  |
| 29615110 | Metagenomics (human) | 199 | N |  |
| 29607983 | Metagenomics (human) | 47 | N |  |
| 26334731 | Metagenomics (human) | 22 | Y | 34 |
| 32082246 | Cross Sectional | 312 | Y | 32 |
| 31928326 | Cross Sectional | 362 | Y | 36 |
| 31337961 | Cross Sectional | 129 | N |  |
| 31167879 | Cross Sectional | Not Available | Y | 27 |
| 31162138 | Cross Sectional | 135 | Y | 11 |
| 31068891 | Cross Sectional | 194, 153 | Y | 33 |
| 30778376 | Cross Sectional | 141 | Y | 9 |
| 30313111 | Cross Sectional | 20 | Y | 30 |
| 29914158 | Cross Sectional | 276 | N |  |
| 29414011 | Cross Sectional | 220 | N |  |
| 29248242 | Cross Sectional | 122 | N |  |
| 28925931 | Cross Sectional | 40 | Y | 17 |
| 28849032 | Cross Sectional | 20 | N |  |
| 28646792 | Cross Sectional | 33 | Y | 35 |
| 28645263 | Cross Sectional | 99 | N |  |
| 28166278 | Cross Sectional | 81 | N |  |
| 27777189 | Cross Sectional | 62 | N |  |
| 26972052 | Cross Sectional | 4007, 11 | N |  |
| 26676906 | Cross Sectional | 227 | Y | 23 |
| 26567910 | Cross Sectional | 1967 | N |  |
| 30721181 | Experimental | 102 | N |  |
