# Supplementary material for: At the Intersection of Gut Microbiome and Stroke: A Systematic Review of the Literature
Source: Front Neurol. 2021 Sep 24;12:729399. doi: 10.3389/fneur.2021.729399 (PMC8498333; doi:10.3389/fneur.2021.729399)
Supplement: Supplementary file 2 [file Table_2.docx]

**Supplemental Table S2.** Data Collection Template.

| PMID | Title | Journal | Year | Study Design | Sample Size | Setting | Stroke Cohort  (y/n)* | Summary | Ref** |
| --- | --- | --- | --- | --- | --- | --- | --- | --- | --- |
| 32189845 | Prevalence of herpesviruses in periodontal disease of the North Indian population: A pilot study. | J Indian Soc Periodontol | 2020 | Case control | 48 cases & 48 controls | Hospital | NO | Gingiva tissue samples were collected from patients with periodontal disease and healthy controls. Almost 20% of the patients had Epstein-Barr virus (EBV) compared to healthy controls, which had no EBV. Thus, periodontal disease is associated with EBV. |  |
| 32188747 | Different Types of Atrial Fibrillation Share Patterns of Gut Microbiota Dysbiosis. | mSphere. | 2020 | Metagenomic and metabolomic analyses | 30 PAF patients, 20 psAF patients, and 50 non-AF controls | Hospital | NO | Gut dysbiosis is involved in atrial fibrillation. Atrial fibrillation subtypes include paroxysmal AF (PAF) and persistent AF (psAF). Alpha diversity was similar between PAF and psAF patients at the genus and species level. These two types of AF have similar taxonomic profiles/microbial structure and it is distinct from the non-AF controls. AF subtypes have similar metabolic profiles in serum and feces. Minor differences between the two AF subtypes include differentially enriched 8 families, 10 genera, and 118 species. Importantly, family Holosporaceae, two genera, and five species showed significant correlation with the atrial diameter. Further, psAF-enriched family Holosporaceae and genus Holospora, in addition to genus Methylovulum and species Methylovulum miyakonense, showed positive correlation with psAF-enriched serum choline. Choline was significantly associated with enlarged atrium. | 13 |
| 32082246 | Dynamic Changes and Prognostic Value of Gut Microbiota-Dependent Trimethylamine-N-Oxide in Acute Ischemic Stroke. | Front Neurol. | 2020 | Cross sectional | 204 AIS pts & 108 controls | Hospital | YES | TMAO levels in AIS patients (thrombotic, lacunar, embolic, other cause, unknown cause) decreased significantly 24 hours after treatment. Baseline TMAO levels are higher in AIS patients than in controls and lower in AIS patients than in controls 7 days after treatment. Antithrombotic agents such as aspirin and clopidogrel decrease TMAO levels. Higher TMAO levels were associated with ischemic events at day 90 or 1 year. Baseline TMAO improved prognostic accuracy of conventional risk factors, NIHSS score, NT-proBNP levels and TMAO levels before treatment had an additive prognostic value. | 32 |
| 31996208 | Quality of whole genome sequencing from blood versus saliva derived DNA in cardiac patients. | BMC Med Genomics. | 2020 | Whole-genome sequencing | 531 blood and 502 saliva samples from cardiac pts | Heart Centre Biobank Registry | NO | Compared to blood derived DNA, higher proportion of saliva derived DNA failed quality control for whole genome sequencing. However, saliva derived DNA that met the quality requirements provided good data and can be used for whole genome sequencing (single nucleotide variants) when blood derived DNA is not available. |  |
| 31956606 | Re-initiation of Oral Food Intake Following Enteral Nutrition Alters Oral and Gut Microbiota Communities. | Front Cell Infect Microbiol. | 2019 | Metagenomics | 8 stroke pts | Hospital | YES | Stroke is associated with dysphagia. 8 sub acute stroke patients on tube feedings had their saliva and fecal samples analyzed before and after re-initiation of oral food intake. The microbes differ in the mouth compared to the gut. The microbes in the mouth and the gut were altered after oral food intake. After oral feeding, there is increased diversity. After oral food intake, the mouth had overrepresented Carnobacteriaceae and Granulicatella, increased proportion of G. adiacens, overrepresented Veillonellaceae, increases in the abundance of phylum Actinobacteria, genus Rothia and Rothia sp., and decreases in that of genus Parvimonas, Parvimonas sp., genus Neisseria, and E. casseliflavus. After oral food intake, the gut had overrepresented Carnobacteriaceae and Granulicatella, increased proportion of G. adiacens, increased Streptococcaceae, genus Streptococcus, and Streptococcus spp (not statistically sig.). Metagenome prediction of mouth microbiota had downregulation of genes associated with immunological disease. The gut had upregulation of genes associated with nucleotide metabolism and had differentially enriched pathways associated with fatty acid metabolism. Oral food intake decreased microbial network in both the mouth and the gut. Lactobacillus salivarius was an interacting core species in the mouth while Christensenella sp. Was an interaction core species in the gut. | 37 |
| 31929168 | The Association of Post-Stroke Cognitive Impairment and Gut Microbiota and its Corresponding Metabolites. | J Alzheimers Dis. | 2020 | Cohort, experimental | 30 AIS PSCI & 35 AIS non-PSCI; 18 AIS given probiotics & 65 AIS controls (newly diagnosed) | Hospital | YES | Post stroke cognitive impairment can lead to disabilities and the gut microbiota is involved in cognitive impairment. 30 PSCI patients and 35 non-PSCI patients had their fecal samples collected and a cognitive assessment performed 3 months later. PSCI patients had less diversity and altered composition of gut microbes compared to non-PSCI patients. The PSCI patients also had increased abundance of Fusobacterium and decreased short chain fatty acids compared to non-PSCI patients. Gut microbiota and SCFA can serve as biomarkers to predict PSCI. Compared to these 65 patients, 18 new AIS (not cerebral hemorrhage) patients were treated with probiotics. Those who received probiotic treatments had improved mood but did not differ in proportion of PSCI. Thus, gut microbes are involved in cognitive impairment. |  |
| 31928326 | Increasing trimethylamine N-oxide levels as a predictor of early neurological deterioration in patients with acute ischemic stroke. | Neurol Res. | 2020 | Cross sectional | 97 AIS END & 265 AIS non-END (first ever AIS) | Hospital | YES | END is early neurological deterioration, which is an increase in NIHSS by 2 or more points from admission up until 3 days. High TMAO is associated with AIS END (thrombotic, lacunar, embolic, other). | 36 |
| 31777426 | Analysis of gut microbiota in patients with cerebral autosomal dominant arteriopathy with subcortical infarcts and leukoencephalopathy (CADASIL). | J Clin Biochem Nutr. | 2019 | Meta16S analysis | 15 CADASIL patients and 16 controls | Outpatient clinic | YES | Cerebral autosomal dominant arteriopathy with subcortical infarcts and leukoencephalopathy (CADASIL) phenotype can be potentially modified by gut microbiota. CADASIL patients and controls as well as CADASIL patients with a history of stroke and CADASIL patients without a history of stroke did not differ in microbial diversity. CADASIL patients had an increase in 6 genera, a decrease in 2 genera, increase in 24 OTUs and decrease in 4 OTUs compared to non-CADASIL patients. CADASIL patients with stroke had a decrease in 2 genera, increase in 13 OTUs, and decrease in 3 OTUs compared to CADASIL patients without stroke. Thus, gut microbiota can modify the onset and progression of CADASIL. | 18 |
| 31620923 | Gastro-intestinal and oral microbiome signatures associated with healthy aging. | Geroscience. | 2019 | Case control | 32 non-healthy & 33 healthy | Hospital | NO | The healthy and non-healthy group had their saliva and fecal samples collected. The healthy group had increased diversity in the oral microbiome and no differences were observed in the gut microbiome between the healthy and the non-healthy group. The healthy group had increased abundance of Akkermansia and Erysipelotrichaceae UCG-003 in the gut microbiome compared to the non-healthy group. The healthy group had decreased abundance of Streptococcus in the oral and gut microbiomes compared to the non-healthy group. These microbes may act as probiotics that promote healthy aging. | 4 |
| 31590480 | Elevation of the Gut Microbiota Metabolite Trimethylamine N-Oxide Predicts Stroke Outcome | J Stroke. | 2019 | Cohort, Cross Sectional | 313 stroke pts (first ever) | Hospital, phone interview | YES | Patients (thrombotic, lacunar, embolic) were placed in two groups: High TMAO level and low TMAO level. During follow up 3 years later, the patients from the high TMAO level group had higher reported major adverse cardiovascular events and occurrences than the low TMAO level group. Thus, TMAO is an independent predictor of MACE in patients with first stroke. | 41 |
| 31426765 | Change of intestinal microbiota in cerebral ischemic stroke patients. | BMC Microbiol. | 2019 | Case control | 30 CI & 30 controls | Hospital | YES | Cerebral ischemic stroke patients and healthy controls did not differ in gut microbial diversity or microbiota structure but did differ in abundance of bacteria. The CI patients had enriched Odoribacter*,*Akkermansia*,*Ruminococcaceae_*UCG_005,*norank_p_Flavobacteriaceae*,*norank_p_Parcubacteria*,* and Victivallis and the healthy controls had enriched Anaerostipes and Ruminiclostridium*_*5. The CI group had increased SCFA producing bacteria, which can potentially be used as biomarkers. | 29 |
| 31420758 | Association of plasma trimethylamine-N-oxide levels with post-stroke cognitive impairment: a 1-year longitudinal study. | Neurol Sci. | 2020 | Cohort | 86 AIS w/ PSCI & 170 AIS w/out PSCI (first ever) | Hospital | YES | AIS pts (thrombotic, lacunar, embolic, other cause) with high TMAO levels had a higher likelihood of developing cognitive impairment 1 year after stroke. TMAO may serve as a biomarker for PSCI. |  |
| 31382927 | Association of Constipation with risk of end-stage renal disease in patients with chronic kidney disease. | BMC Nephrol. | 2019 | Cohort | 26117 | Taiwan National Health Insurance database | NO | 9.5% of constipated pts with CKD experienced ESRD compared to 4.7% of non-constipated pts with CKD. Over 13 years, incidence of ESRD was higher in the constipated group compared to the non-constipated group. While mild constipation with no laxative use did not increase ESRD, obvious constipation with laxative use more than one month/year increased the risk of ESRD. Similarly, severe constipation with laxative use greater than half a year had a higher risk of ESRD. CKD and constipation show a dose-effect relationship. |  |
| 31337961 | Differential Analysis of Hypertension-Associated Intestinal Microbiota. | Int J Med Sci. | 2019 | Cross Sectional | 62 HTN cases & 67 normal BP cases | Hospital | NO | Hypertension is a risk factor for stroke. Fecal samples from patients with high blood pressure and normal blood pressure was examined. There were 54 differential genera between these two groups. There were 5 differential genera between the normal blood pressure group and the isolated diastolic hypertension group. There were 38 differential genera between the normal blood pressure group and the systolic hypertension group. The blood pressure correlates with gut microbial abundance. |  |
| 31332666 | Prognostic Value of Plasma Trimethylamine N-Oxide Levels in Patients with Acute Ischemic Stroke. | Cell Mol Neurobiol. | 2019 | Prospective cohort | 225 AIS | Hospital, telephone, outpatient visits | YES | Plasma TMAO was measured for AIS patients (thrombotic, lacunar, embolic, other cause, unknown cause) and functional outcome was determined 3 months later. The highest quartile of serum TMAO was a prosnostic marker for unfavorable functional outcomes (defined as 3-6 on the Rankin Scale). The optimal TMAO level for predicting unfavorable functional outcomes is 3.9 uM and for mortality is 4.0 uM. |  |
| 31308384 | Establishment and evaluation of prediction model for multiple disease classification based on gut microbial data. | Sci Rep. | 2019 | Metanalysis | 1079 individuals who are healthy or have disease including: MS, JIA, ME/CFS, AIDS, CRC, and Stroke | European Bioinformatics Institute (EBI) database | YES | Highest performance for machine learning in disease classification improves with use of lower taxonomy features. |  |
| 31216010 | Duration and life-stage of antibiotic use and risk of cardiovascular events in women. | Eur Heart J. | 2019 | Longitudinal cohort | 1056 CVD & 35373 non-CVD | Questionaires | YES | Female nurses in 3 age groups (20–39, 40–59, 60+) filled out questionaires every 2 years up to 8 years total about their antibiotic use. The antibiotic use was grouped as the following: none, <15 days, 15 days to <2 months, 2 months or more. The most common indications for using antibiotics include respiratory infections, UTI, and dental. Longer duration of antibiotics in middle and older aged groups was associated with CVD (stroke & CHD) |  |
| 31167879 | Assessment of Causal Direction Between Gut Microbiota-Dependent Metabolites and Cardiometabolic Health: A Bidirectional Mendelian Randomization Analysis. | Diabetes. | 2019 | Cross Sectional | NA | Databases | NO | Genetically predicted higher TMAO or carnitine was not associated with higher odds of T2DM, AF, CAD, MI, stroke, and CKD. However, genetically increased choline was associated with a greater risk of T2DM but not CKD or cardiovascular events. Higher betaine was associated with lower risk of T2DM. Further, T2DM and CKD was causally associated with higher TMAO levels. | 25 |
| 31164901 | Multi-Omic Analysis of the Microbiome and Metabolome in Healthy Subjects Reveals Microbiome-Dependent Relationships Between Diet and Metabolites | Front Genet. | 2019 | Multi-Omic Analysis | 150 healthy individuals free of illness, consuming american diet | University | NO | The gut microbiome is related to diet and metabolites. Short term diet is significantly associated with gut and plasma metabolome but not microbiome. Long term diet. Long term diet is associated with gut and plasma metabolome as well as strongly associated with gut microbiome. Gut microbiome is strongly associated with gut metabolome. 61 long-term dietary nutrients are associated with at least one bacterial genus. Some nutrients were associated with 3 or more genera; these nutrients are found in plant based diets and dairy foods. 123 plasma metabolites and 34 gut metabolites were associated with at least one bacterial genus. These metabolites are involved in bile acid metabolism, lipid and amino acid metabolism, or metabolism of xenobiotics. There were 2 enterotypes: Individuals with enterotype 2 had lower BMI and a higher proportion of Ruminococcaceae. There were differences in 112 plasma metabolites and 122 stool metabolites between the 2 enterotypes. |  |
| 31162138 | High dietary salt-induced dendritic cell activation underlies microbial dysbiosis-associated hypertension. | JCI Insight. | 2019 | Cross Sectional | 135 healthy individiuals | University | NO | Individuals on a low-salt or high-salt diet have similarities in α diversity. However, there is a significant difference in β diversity between short-term high vs low salt intake. High salt intake (≥2.3 g/d) was associated with increased relative abundance of bacterial taxa, including genus Prevotella, family Ruminococcaceae, and genus Bacteroides. Further, individuals with short term high salt intake had significantly higher systolic and diastolic blood pressures compared to individuals with long term high salt intake, who had significantly higher systolic blood pressure. There was a positive correlation between high systolic blood pressure and Prevotella. Hypertension is associated with gut pathology (arteriolar wall thickening and fibrosis), inflammation (T cells and macs in intestinal wall), and acculumaltion of IsoLG in colon compared to individuals with normal blood pressure. | 11 |
| 31151471 | Dysbiosis of the intestinal microbiota in neurocritically ill patients and the risk for death. | Crit Care. | 2019 | Prospective observational cohort | 98 neurocritically ill pts (including 38 ischemic stroke & 20 intracerebral haemorrhage) & 84 controls | Hospital, community | YES | The gut microbiota in 58 stroke patients (ischaemic stroke and intracerebral haemorrhage) are different from 58 healthy controls, including β-diversity, α-diversity, taxonomic summary and LEfSe analysis. | 28 |
| 31123156 | Utility of Plasma Concentration of Trimethylamine N-Oxide in Predicting Cardiovascular and Renal Complications in Individuals With Type 1 Diabetes. | Diabetes Care. | 2019 | Cohort | 1159 diabetic pts | Outpatient clinic | YES | Type 1 diabetic patients with higher plasma TMAO levels have higher risk of mortality, cardiovascular events, and poor renal outcomes (ESRD & nephropathy). These associations become insignificant after adjusting for baseline eGFR. High plasma TMAO in type 1 diabetic pts is associated with low baseline eGFR. | 26 |
| 31068891 | Stroke Dysbiosis Index (SDI) in Gut Microbiome Are Associated With Brain Injury and Prognosis of Stroke. | Front Neurol. | 2019 | Cross Sectional | 104 AIS & 90 healthy controls; 83 patients with acute ischemic stroke & 70 healthy individuals (validation group) | Hospital | YES | Stroke dysbiosis index was created for AIS patients (large-artery atherosclerotic ischemic stroke) based on 18 differential genera. SDI correlates with stroke severity (NIHSS) and outcome at discharge (mRS) at discharge. SDI and WBC are independent predictor of severe stroke and SDI is an independent predictor of early unfavorable outcome. | 33 |
| 31049589 | Trimethyllysine, a trimethylamine N-oxide precursor, provides near- and long-term prognostic value in patients presenting with acute coronary syndromes. | Eur Heart J. | 2019 | Prospective cohort | 530 pts w/ chest pain & acute coronary syndrome & 1683 pts w/ acute coronary syndrome | Hospital | YES | TML is a precursor to TMAO. Plasma TML in patients with chest pain and suspected acute coronary syndrome is associated with major cardiac events, including stroke over 30 days and 6 months (independent of CV risk factors or renal function). Increased TML associated with 7 year mortality and MACE in pts negative for cardiac troponin T. TMAO has an additive effect along with traditional risk factors and renal function. | 24 |
| 30873501 | The effects of trans-resveratrol on insulin resistance, inflammation, and microbiota in men with the metabolic syndrome: A pilot randomized, placebo-controlled clinical trial. | J Clin Transl Res. | 2018 | Clinical trial | 28 obese men w/ MetS | Hospital | NO | Metabolic syndrome can result in stroke. Polyphenol resveratrol (RES) activates sirtuin and can improve glucose homeostasis and insulin resistance. RES 2 g given to obese individuals with MetS marginally improved glucose homeostasis. However, Caucasians had their glucose homeostasis and insulin resistance improve compared to non-Caucasians. RES reduced microbial diversity and RES-treated Caucasians had a significant increase in A. muciniphila, which is inversely associated with obesity and diabetes. | 16 |
| 30778376 | Higher Risk of Stroke Is Correlated With Increased Opportunistic Pathogen Load and Reduced Levels of Butyrate-Producing Bacteria in the Gut. | Front Cell Infect Microbiol. | 2019 | Cross sectional correlation | 141 people without history of stroke or MI | Questionaires, Hospital clinical laboratory | YES | Individuals with a high risk of stroke had increased gut opportunistic pathogens and lactate producing bacteria compared to low risk individuals, with the greater difference in Enterobacteriaceae. High risk of stroke was associated with reduced butyrate-producing bacteria and fecal butyrate concentrations. The gut microbiome features in different risk groups is not affected by medication such as statins, antihypertensive drugs, and hypoglycemic drugs. | 9 |
| 30721181 | N/A | N/A |  | Experimental | 102 | N/A | NO | N/A |  |
| 30658186 | Constipation and risk of death and cardiovascular events. | Atherosclerosis. | 2019 | Cohort | 237,855 constipated pts & 3,121,798 non-constipated pts | Department of Veteran Affairs health care facilities | YES | Constipated pts had a 12% higher risk of mortality, 11% higher risk of CHD and 19% higher risk of ischemic stroke compared to those without constipation. Constipation is independenty associated with incident CHD, ischemic stroke, mortality. Patients using laxatives had a similar higher risk of mortality, CHD, and stroke. |  |
| 30553164 | Gut microbiota-dependent trimethylamine-N-oxide (TMAO) shows a U-shaped association with mortality but not with recurrent venous thromboembolism. | Thromb Res. | 2019 | Prospective cohort | 859 elderly pts with acute venous thromboembolism | Multicenter including hospital, laboratory, phone interview | NO | Elderly pts with venous thromboembolism had their plasma TMAO levels measured and were followed up for 28 months to observe associations between VTE and mortality, bleeding, recurent VTE. TMAO was not associated with recurrent VTE or bleeding. TMAO in elderly pts with VTE has a U shaped association with mortality with 4 microMolar/L of TMAO associated with lowest risk of death. |  |
| 30354996 | Serum Trimethylamine N-Oxide Concentration Is Positively Associated With First Stroke in Hypertensive Patients. | Stroke | 2018 | Nested case-control | 622 pts w/ first stroke & 622 controls | Data from a randomized, double blind clinical trial | YES | Baseline serum TMAO in hypertensive patients without a history of stroke, MI, or other cardiovascular diseases were measured to determine an association with first stroke within a 4.5 year followup. Higher TMAO in hypertensive pts is associated with first stroke (ischemic or hemorrhagic, not subarachnoid hemorrhage or silent stroke) | 25 |
| 30313111 | The characteristics analysis of intestinal microecology on cerebral infarction patients and its correlation with apolipoprotein E. | Medicine (Baltimore). | 2018 | Cross Sectional | 10 CI pts & 10 controls | Hospital | YES | Cerebral infarction (CI) pts had an increase in Gammaproteobacteria and decrease in Bacteroidia compared to controls. ApoE was negatively correlated with Bacteroidia and positively correlated with Gammaproteobacteria. These microbes may induce ApoE/blood lipids to lead to the development of CI. | 30 |
| 30100156 | Moderate Renal Impairment and Toxic Metabolites Produced by the Intestinal Microbiome: Dietary Implications. | J Ren Nutr. | 2019 | Cohort | 316 pts | Academic medical center | YES | Pts had their plasma levels of gut-derived uremic toxins (GDUT), including TMAO and p-cresyl sulfate (PCS) among others, measured as well as their kidney function measured. Nutrient intake over the past year was reported by patients on questionaires. Lower quartile of eGFR had higher plasma TMAO and moderate renal impairment was associated with higher GDUT. Dietary precursors can predict PCS in patients with impaired renal function and dietary precursors can predict TMAO in patients with eGFR greater than 60. eGFR is a predictor for metabolites. |  |
| 29976769 | Gut Microbiota-Dependent Trimethylamine N-Oxide Predicts Risk of Cardiovascular Events in Patients With Stroke and Is Related to Proinflammatory Monocytes. | Arterioscler Thromb Vasc Biol. | 2018 | Prospective cohort | 78 first ever ischemic stroke pts; 593 first ever stroke | Hospital | YES | In patients with first ever stroke, TMAO levels, not its precursor choline, has a dose dependent relationship with 1 year cardiovascular events including recurrent stroke, MI, death. The validation cohort showed that TMAO levels are associated with 1 year combined CVE (recurrent stroke, MI, death) and also recurrent stroke alone. TMAO has a prognostic value. The proportion of intermediate CD14++CD16+ monocytes has a dose-dependent increase according to TMAO levels. |  |
| 29930110 | Analysis of shared heritability in common disorders of the brain. | Science | 2018 | Comprehensive heritability and correlation analysis | 265,218 pts & 784,643 control | Genome wide association data | NO | Genome-wide common variant risk between 2 individuals can be measured to determine how connected the phenotypes are at the genomic level. |  |
| 29914158 | Mediterranean Diet Score: Associations with Metabolic Products of the Intestinal Microbiome, Carotid Plaque Burden, and Renal Function. | Nutrients. | 2018 | Cross-sectional | 276 high risk vascular pts with severe atherosclerosis | Clinical database included pts from clinics | NO | High risk vascular pts had their plasma GDUT measured, carotid plaque burden measured, and also filled out a questionaire about their diet. Moderate renal impairment increased plasma GDUT and the increase in GDUT was associated with higher carotid plaque burden. In omnivore pts, nutrient precursors or Mediterrarean diet does not predict plasma metabolites; thus, the microbiome may be responsible instead of diet. |  |
| 29900345 | Data on the gut and saliva microbiota from a cohort of atherosclerosis patients determined by 16S rRNA gene sequencing | Data Brief. | 2018 | Metagenomics | 20 volunteers | NA | NO | Pts with traditional risk factors for atherosclerosis were placed into two groups: expected to get atherosclerosis but did not & not expected to get atherosclerosis & did. Their saliva and stool samples were collected and characterized using OTUs. |  |
| 29702430 | Metabolic products of the intestinal microbiome and extremes of atherosclerosis | Atherosclerosis | 2018 | Cohort, Cross Sectional | 316 | Clinic | NO | Intestinal microbiome metabolic products play a role in carotid atherosclerosis. |  |
| 29678946 | Prenatal antibiotic exposure and childhood asthma: a population-based study. | Eur Respir J | 2018 | Retrospective population-based cohort | 213,661 mother–child dyads | Health administrative data | NO | Antibiotic use in pregnant mothers and development of asthma in child was examined. Children born to mothers receiving antibiotics during pregnancy had significantly higher rates of asthma (11.9 per 1000 person-years) compared to their unexposed counterparts (9.2 per 1000 person-years). Asthma risk increases with increased exposure of antibiotics. The type of antibiotics, including beta lactam penicillin and macrolides, were similarly associated with child asthma. Antibiotic use during first, second, or third trimester were similarly associated with child asthma. Antibiotic exposure to pregnant women was associated with a 23% increased risk of asthma in child, independent of postnatal antibiotic exposure and asthma risk factors. |  |
| 29615110 | Gut-dependent microbial translocation induces inflammation and cardiovascular events after ST-elevation myocardial infarction. | Microbiome | 2018 | Metagenomic analysis | 49 healthy controls, 50 stable CHDs, and 100 STEMI pts | Hospital | NO | Gut micrbiota translocation into circulation impacts post-myocardial infaction outcomes. |  |
| 29607983 | Non-Ischemic Heart Failure With Reduced Ejection Fraction Is Associated With Altered Intestinal Microbiota | Circ J | 2018 | Metagenomic | Twenty eight non-ischemic HFrEF patients and 19 healthy controls | Hospital | NO | There exists microbiota differences between healthy patients and individuals with non-ischemic heart failure with reduced ejection fraction. |  |
| 29414011 | Simultaneous determination of trimethylamine N-oxide, choline, betaine by UPLC-MS/MS in human plasma: An application in acute stroke patients. | J Pharm Biomed Anal | 2018 | NA | 220 acute ischemic stroke | Hospital | YES | The UPLC–MS/MS method was developed to quantify TMAO, choline, betaine in human plasma. Blood samples were taken from AIS pts after they received antiplatelet therapy. Choline was associated with mRS, which can be a biomarker to predict prognosis of AIS pts. Elevated plasma choline were associated with unfavorable outcomes at 6 months. |  |
| 29248242 | Plasma microbiome-modulated indole- and phenyl-derived metabolites associate with advanced atherosclerosis and postoperative outcomes | J Vasc Surg | 2018 | Cohort | 100 atherosclerosis & 22 control | University | NO | There exists biomarkers that include tryptophan associated with atherosclerosis and cardiovascular complications. |  |
| 29080862 | Plasma Metabolites From Choline Pathway and Risk of Cardiovascular Disease in the PREDIMED (Prevention With Mediterranean Diet) Study. | J Am Heart Assoc. | 2017 | Case control | 229 incident CVD cases & 751 randomly selected participants at baseline | N/A | YES | Products of choline pathway are associated with increased cardiovascular disease. |  |
| 28925931 | Gut Microbiota-Dependent Trimethylamine-N-oxide and Serum Biomarkers in Patients with T2DM and Advanced CKD. | J Clin Med | 2017 | Cross sectional | 20 T2DM-CKD pts & 20 healthy controls | Outpatient clinic | NO | Pts had an increased abundance in TMA producers (Clostridium, Escherichia, Enterobacter, Acinetobacter, and Proteus) that belong to the phylum Firmicutes and Proteobacteria in gut microbiome. This study identified 8 more bacterial genera belonging to 2 phyla, Firmicutes and Proteobacteria, which was associated with production of TMA from choline and carnitine. Pts also have increased TMAO. Pts had increased serum markers for TNFa, IL-6, zonulin (gut permeability), ET-1 (endothelial dysfunction), LPS. TMAO showed correlation with TNFa, IL-6, zonulin, ET-1, LPS. | 17 |
| 28849032 | Analysis of intestinal microbial communities of cerebral infarction and ischemia patients based on high throughput sequencing technology and glucose and lipid metabolism | Mol Med Rep. | 2017 | Cross sectional | 8 CI pts, 2 IS pts & 10 healthy controls | Hospital | YES | CI had a difference in abundance of gut bacteria at the phylum level compared to controls; this difference was not observed when comparing IS to controls. At the genus level, both CI & IS had difference in abundance of gut bacteria compared to controls. Also, CI & IS pts had difference in abundance of gut bacteria at the genus level compared to each other. Blood glucose levels in CI & IS pts was higher than healthy controls. |  |
| 28646792 | Bacterial profile in human atherosclerotic plaques. | Atherosclerosis. | 2017 | Cross sectional | 11 asymptomatic atherosclerosis (TIA or amaurosis fugax) and 22 symptomatic atherosclerosis (stroke) pts undergoing carotid endarterectomy | Hospital | NO | Study found that there is bacterial DNA in atherosclerotic plaque samples, dominated by Proteobacteria and Actinobacteria. The bacterial profile is similar between symptomatic (cerebral ischemia including stroke, TIA, or amaurosis fugax (AFX)) and asymptomatic pts. The amount and type of bacterial DNA in atherosclerotic plaques were similar along the length of the plaque. | 35 |
| 28645263 | Microbiota-dependent metabolite and cardiovascular disease marker trimethylamine-N-oxide (TMAO) is associated with monocyte activation but not platelet function in untreated HIV infection | BMC Infect Dis | 2017 | Cross-sectional | 50 untreated and 49 cART treated HIV-infected individuals. | Hospital | NO | TMAO does not induce platelet hyperreactivity in HIV-infected pts. Inflammatory effects may impact TMAO levels in untreated HIV pts. |  |
| 28543778 | Oral flora in acute stroke patients: A prospective exploratory observational study. | Gerodontology | 2017 | Cohort | 50 acute stroke pts | Hospital | YES | There exists an abundance of oral bacterial flora in post-stroke patients. |  |
| 28407784 | Relationships between gut microbiota, plasma metabolites, and metabolic syndrome traits in the METSIM cohort. | Genome Biol. | 2017 | Population-based cohort | 531 individuals (middle aged men) | Metabolic Syndrome in Men (METSIM) cohort | YES | In men with metabolic syndrome, gut microbiota is associated with fasting serum levels of fatty acids, amino acids, lipids, and glucose. "51 OTUs were significantly associated with fatty acids, 33 OTUs with ketone bodies, 19 OTUs with amino acids and glycolysis-related metabolites, nine OTUs with glycoprotein acetyls, six OTUs with choline pathway metabolites, and three OTUs with HbA1c levels. At taxonomy level, 19 taxa were associated with fatty acids, nine with ketone bodies, five with glycolysis-related metabolites, three with amino acids, two with glycoprotein acetyls, and one each with betaine and TMAO." Pasma TMAO concentrations significantly associated with Prevotella and Peptococcaceae. Plasma TMAO concentrations had a negative correlation with F. prausnitzii. | 14 |
| 28166278 | Gut dysbiosis is associated with metabolism and systemic inflammation in patients with ischemic stroke. | PLoS One. | 2017 | Cross sectional | 41 ischemic stroke patients and 40 control | Hospital | YES | Ischemic stroke is associated with dysbiosis and inflammatory alterations. |  |
| 28077467 | Gut microbiota-dependent trimethylamine N-oxide in acute coronary syndromes: a prognostic marker for incident cardiovascular events beyond traditional risk factors | Eur Heart J | 2017 | Cohort | 530, 1683 | University | YES | In patients presenting with chest pain, plasma levels of TMAO can predict cardiovascular events. |  |
| 27777189 | N/A | N/A | N/A | Cross sectional | 62 | N/A | YES | N/A |  |
| 27515213 | Vitamin D deficiency changes the intestinal microbiome reducing B vitamin production in the gut. The resulting lack of pantothenic acid adversely affects the immune system, producing a "pro-inflammatory" state associated with atherosclerosis and autoimmunity. | Med Hypotheses. | 2016 | Cohort | >1000 neurology pts such as headache, vertigo, epilepsy, tics, pain, multiple sclerosis, neuropathy, Parkinson’s disease, tremor, dystonia, cerebellar degeneration, depression, fatigue, memory loss, insomia, sleep anea (not brain cancer, amyotrophic lateral sclerosis, or Alzheimer’s disease) | N/A | NO | Vitamin D promotes normal sleep in neurology patients. Vitamin D and B100 given for 3 months to neurology pts resulted in improved sleep and bowel symptoms (IBS, constipation). |  |
| 26972052 | Gut microbial metabolite TMAO enhances platelet hyperreactivity and thrombosis risk | Cell. | 2017 | Cross sectional | > 4000 healthy individuals | NA | YES | In healthy individuals, TMAO was associated with 3 year incident thrombosis risk (including MI and stroke) after adjusting for CVD, traditional CVD risk factors, renal function and medication use. TMAO directly enhanced human platelet function and responsiveness to agonists such as ADP, thrombin and collagen. |  |
| 26868510 | The Carnitine-butyrobetaine-trimethylamine-N-oxide pathway and its association with cardiovascular mortality in patients with carotid atherosclerosis. | Atherosclerosis. | 2016 | Cohort | 264 pts w/ moderate to severe carotid stenosis & 62 healthy controls | Hospital | NO | γ-butyrobetaine (γBB) is a carnitine-derived metabolite. Pts with carotid atherosclerosis have higher serum yBB and carnitine but not TMAO or TML. γBB and TML were independently associated with cardiovascular death, after adjusting for age and eGFR. |  |
| 26731277 | Irritable Bowel Syndrome Is Associated with an Increased Risk of Dementia: A Nationwide Population-Based Study. | PLoS One. | 2016 | Population-based cohort | 32 298 adult patients with IBS & 129 192 patients without IBS. | Database | NO | IBS pts had increased risk of dementia after adjusting for age, sex, diabetes, hypertension, stroke, CAD, head injury, depression, and epilepsy. IBS was associated with an increased risk of dementia in patients older than 50 years in both male and female. |  |
| 26676906 | Associations among serum trimethylamine-N-oxide (TMAO) levels, kidney function and infarcted coronary artery number in patients undergoing cardiovascular surgery: a cross-sectional study. | Clin Exp Nephrol. | 2016 | Cross sectional | 227 patients who underwent cardiovascular surgery for coronary artery disease, valvular heart disease, or aortic disease | ICU of university medical center | NO | In pts who had cardiovascular surgery, high serum TMAO was associated with higher number of infarcted coronary arteries, after adjusting for age, sex, BMI, CKD stage, hypertension, DLP, cerebrovascular disease. Higher quartile of TMAO had higher number of infarcted coronary arteries, independent of CAD risk factors. TMAO levels were increased in patients advanced CKD. | 23 |
| 26597155 | Dysbiosis of Gut Microbiota With Reduced Trimethylamine-N-Oxide Level in Patients With Large-Artery Atherosclerotic Stroke or Transient Ischemic Attack. | J Am Heart Assoc. | 2015 | Case control | 322 large‐artery atherosclerotic ischemic stroke and TIA pts & 231 asymptomatic controls | Hospital | YES | Large‐artery atherosclerotic ischemic stroke and TIA pts had alterations in their gut, including depleted Bacteroides, Prevotella, and Faecalibacterium (good bacteria) and increased Enterobacter, Megasphaera, and Desulfovibrio (opportunistic bacteria). Proteobacteria is abundant in severe stroke patients compared with mild stroke patients. Blood TMAO was lower in stroke & TIA pts. Asymptomatic atherosclerotic pts did not have alterations in gut dysbiosis or TMAO levels. | 31 |
| 26567910 | Effect of renal impairment on atherosclerosis: only partially mediated by homocysteine. | Nephrol Dial Transplant. | 2016 | Cross sectional | 1967 patients | Clinical database of the Stroke Prevention & Atherosclerosis Research Centre, | NO | With age, eGFR decreases and carotid total plaque area as well as carotid stenosis increases linearly. |  |
| 26536303 | Oral microbiota in patients with atherosclerosis. | Atherosclerosis. | 2015 | Case control | 62 pts & 30 controls | Hospital | YES | Microbiota may contribute to atherosclerosis. Periodontal disease is associated with cerebrovascular and cardiovascular events. Here, oral microbiota was analyzed between patients with atherosclerosis and controls. Microbial composition was similar between patients and controls at the phylum level. However, at the genus level, symptomatic patients had an abundance of Anaeroglobus compared to the controls. Thus, Anaeroglobus in the oral cavity could be associated with symptomatic atherosclerosis. Also, association between bacteria and cardiovascular disease revealed that Parvimonas was associated with uCRP (inflammation) and Capnocytophaga, Catonella and Lactobacillus were associated with blood lipid markers. |  |
| 26413854 | Microbiota-Dependent Marker TMAO Is Elevated in Silent Ischemia but Is Not Associated With First-Time Myocardial Infarction in HIV Infection. | J Acquir Immune Defic Syndr. | 2016 | Cross-sectional & nested case control | 105 HIV pts & 105 controls; 51 HIV pts w/ MI & 164 controls w/ HIV | Outpatient, University hospital | NO | HIV is associated with coronary heart disease and an altered gut microbiota may be a trigger. First study: HIV patients and their controls had their blood samples collected. Betaine was lower in HIV patients compared to controls and TMAO and choline levels were similar between these two groups. HIV patients with myocardial perfusion defects had increased TMAO levels compared to HIV patients without MPD. Second study: HIV patients with MI and HIV controls without MI had their blood collected. TMAO, choline, and betaine levels were similar between these two groups. This shows that TMAO is not associated with first time MI. After the introduction of ART therapy (PI) in both cases and controls, TMAO levels increased. |  |
| 26334731 | In silico analyses of metagenomes from human atherosclerotic plaque samples | Microbiome. | 2015 | Comparative metagenomic analysis | 15 pts that had carotid endarterectomy from previous strokes or TIA & 7 asymptomatic atherosclerotic pts who died not due to athersoclerosis | NA | YES | Microbial infection promotes cardiovascular disease. Microbial communities leading to atherosclerosis was investigated by comparing patients who had carotid endarectomy due to previous strokes or TIA and asymptomatic atherosclerotic patients. The asymptomatic plaques had an abundance of Porphyromonadaceae, Bacteroidaceae, Micrococcaceae, and Streptococcaceae and the symptomatic plaques had an abundance of Helicobacteraceae and Neisseriaceae as well as Acinetobacter, Acidovorax, and N. polysaccharea. Thus, asymptomatic and symptomatic patients have differences in microbial communities in plaques. | 34 |
| 25522313 | Serum IgG antibody levels to periodontal microbiota are associated with incident Alzheimer disease. | PLoS One. | 2014 | Cohort | 110 incident AD cases and 109 controls without incident cognitive impairment at last follow-up (eldery individuals with no cognitive impairment or dementia at the start of the study) | Washington Heights Inwood Columbia Aging Project (WHICAP) | NO | Periodontitis and Alzheimer disease are associated with inflammation. Serum IgG antibodies to periodontal pathogens (which had positive or negative associations with periodontitis) were asessed as risk factor for AD. Individuals with higher A. naeslundii serum IgG had higher risk for incident AD, after adjusting for age, vascular risk factors, DM, stroke, and hypertension. Similarly, elevated E. nodatum serum IgG (>1755 ng/ml) had a decreased risk of incident AD after adjusting for variables. |  |
| 24497336 | Prognostic value of choline and betaine depends on intestinal microbiota-generated metabolite trimethylamine-N-oxide. | Eur Heart J. | 2014 | Prospective population based cohort | 3903 | Clinic | YES | Higher plasma choline or betaine were associated with increased risk for MACE within 3 years in humans with increased TMAO levels (after adjusting for traditional CVD risk factors). Mice fed choline had increased plasma TMAO levels. Mice fed betaine had increased plasma TMAO levels. Antibiotic use showed that TMAO production from betaine is dependent on gut flora. |  |
| 23614584 | Intestinal microbial metabolism of phosphatidylcholine and cardiovascular risk | N Engl J Med. | 2013 | Cohort, Experimental | 4007, 40 | Laboratory | YES | First study: Healthy individuals were given a phosphatidylcholine challenge (egg yolk and a tracer) and choline metabolites were measured. Fasting plasma at baseline had TMAO, choline, betaine present. After the challenge, TMAO, choline, betaine levels increased in the (time dependent changes) plasma and urine. Antibiotics suppressed TMAO levels and discontinuation of antibiotics brought back the TMAO levels. Second study: Individuals undergoing a cardiac catheterization and had no coronary syndrome had their fasting blood samples collected. After a 3 year follow up, increased fasting plasma TMAO levels were associated with MACE, even after adjusting for risk factors. |  |
| 20453665 | Periodontal bacteria and hypertension: the oral infections and vascular disease epidemiology study (INVEST). | J Hypertens. | 2010 | Prospective population based cohort | 653 participants w/ no Hx of stroke, MI, chronic inflammatory conditions | NA | NO | Periodontal infection can lead to cardiovascular diseases such as hypertension. Individuals had their subgingival plaques collected and their blood pressure taken. This study found an association between subgingival colonization by A. actinmycetemcomitans, P. gingivalis, T. forsythia and T. denticola (etiologic burden) and hypertension. This association was stronger in men than in women. Individuals with periodontal disease related pathogens had increased blood pressure (both systolic and diastolic) and prevalent hypertension after adjusting for conventional risk factors. |  |
| 19053919 | Similarities in the subgingival microbiota assessed by a curet sampling method at sites with chronic periodontitis. The 37 species that are studied for periodontitis have similar bacterial count among molar, premolar, and incisor sites. | J Periodontol. | 2008 | Cohort | 161 individuals w/ periodontitis & had subgingival debridement 6 months ago | Database | NO | Pts with periodonal disease had their subgingival samples collected from 6 different sites via steril curettes. 37 species commonly studied for periodontitis were similar in bacterial count in the molar, premolar, and incisor teeth. |  |
| 15699278 | Periodontal microbiota and carotid intima-media thickness: The Oral Infections and Vascular Disease Epidemiology Study (INVEST) | Circulation. | 2005 | Prospective population based cohort | 657 participants w/ no Hx of stroke, MI, chronic inflammatory conditions | University | NO | Periodontal disease is associated with cardiovascular diseases, including atherosclerosis. Subgingival plaques were collected from individuals and they had their carotid IMT assessed. Individuals with periodontal disease related oral pathogens had thicker carotid IMT after adjusting for conventional risk factors (independent of CRP). |  |

*We define the “stroke cohort” column with regards to articles that included stroke patients in the selection criteria, articles with stroke as an outcome, and articles with patients undergoing stroke prevention.

** ref. are only included for studies that were part of this systematic review. The PubMed ID (PMID) is provided for all the studies.
